# A Matter of Timing—Pregnancy After Bariatric Surgery

**DOI:** 10.1007/s11695-020-05219-3

**Published:** 2021-01-11

**Authors:** Laura Heusschen, Ineke Krabbendam, Jessika M. van der Velde, Laura N. Deden, Edo O. Aarts, Ashley E. R. Merién, Marloes Emous, Gysèle S. Bleumink, Helen L. Lutgers, Eric J. Hazebroek

**Affiliations:** 1 Department of Bariatric Surgery, Vitalys, part of Rijnstate hospital, Postal number 1191, PO box 9555, 6800TA Arnhem, The Netherlands; 2 Divison of Human Nutrition and Health, Wageningen University, Wageningen, The Netherlands; 3 Department of Obstetrics and Gynecology, Hospital Gelderse Vallei, Ede, The Netherlands; 4 Department of Obstetrics and Gynecology, Rijnstate hospital, Arnhem, The Netherlands; 5 Center of Obesity the Northern Netherlands, Medical Centre Leeuwarden, Leeuwarden, The Netherlands; 6 Department of Internal Medicine, Rijnstate hospital, Arnhem, The Netherlands; 7 Department of Internal Medicine, Medical Centre Leeuwarden, Leeuwarden, The Netherlands

**Keywords:** Gestational weight gain, Neonatal outcomes, One anastomosis gastric bypass, Roux-en-Y gastric bypass, Sleeve gastrectomy, Surgery-to-conception time interval, NAM, IOM

## Abstract

**Purpose:**

Current guidelines recommend to avoid pregnancy for 12–24 months after bariatric surgery because of active weight loss and an increased risk of nutritional deficiencies. However, high-quality evidence is lacking, and only a few studies included data on gestational weight gain. We therefore evaluated pregnancy and neonatal outcomes by both surgery-to-conception interval and gestational weight gain.

**Materials and Methods:**

A multicenter retrospective analysis of 196 singleton pregnancies following Roux-en-Y gastric bypass, sleeve gastrectomy, and one anastomosis gastric bypass was conducted. Pregnancies were divided into the early group (≤ 12 months), the middle group (12–24 months), and the late group (> 24 months) according to the surgery-to-conception interval. Gestational weight gain was classified as inadequate, adequate, or excessive according to the National Academy of Medicine recommendations.

**Results:**

Pregnancy in the early group (23.5%) was associated with lower gestational age at delivery (267.1 ± 19.9 days vs 272.7 ± 9.2 and 273.1 ± 13.5 days, *P* = 0.029), lower gestational weight gain (− 0.9 ± 11.0 kg vs + 10.2 ± 5.6 and + 10.0 ± 6.4 kg, *P* < 0.001), and lower neonatal birth weight (2979 ± 470 g vs 3161 ± 481 and 3211 ± 465 g, *P* = 0.008) than pregnancy in the middle and late group. Inadequate gestational weight gain (40.6%) was associated with lower gestational age at delivery (266.5 ± 20.2 days vs 273.8 ± 8.4 days, *P* = 0.002) and lower neonatal birth weight (3061 ± 511 g vs 3217 ± 479 g, *P* = 0.053) compared to adequate weight gain. Preterm births were also more frequently observed in this group (15.9% vs 6.0%, *P* = 0.037).

**Conclusion:**

Our findings support the recommendation to avoid pregnancy for 12 months after bariatric surgery. Specific attention is needed on achieving adequate gestational weight gain.

## Introduction

More than half of all female patients undergoing bariatric surgery are of reproductive age. Weight loss after bariatric surgery not only improves fertility [1] but also reduces the risk of gestational diabetes (GDM), hypertensive disorders, and large-for-gestational-age (LGA) neonates [2, 3]. On the other hand, infants born after maternal bariatric surgery may be at risk for preterm birth, admission to the neonatal intensive care unit, and being small-for-gestational-age (SGA) [3–5]. These risks may be most pronounced in pregnancies within the first 12 months after surgery as this period theoretically carries the highest risk of malnutrition due to a markedly reduced caloric intake and rapid weight loss [6]. As a result, nutritional supply to the growing fetus may be decreased. Moreover, maternal caloric restriction and subsequent weight loss during this catabolic period may limit gestational weight gain. In 2009, the National Academy of Medicine (NAM; formerly called the Institute of Medicine) presented recommendations on gestational weight gain according to the women’s pregestational BMI [7]. In overweight and obese women, gestational weight gain below the lower limit of 5 kg is associated with an increased risk of SGA neonates and decreased neonatal birth weight, fat mass, lean mass, birth length, and head circumference [8, 9].

Several organizations have proposed recommendations on the timing of pregnancy following bariatric surgery, but uniformity and scientific evidence are lacking. According to the American Association of Clinical Endocrinology, the Obesity Society, and the American Society for Metabolic and Bariatric Surgery, pregnancy should be avoided for 12–18 months following bariatric surgery (2013) [10] whereas the American College of Obstetricians and Gynecologists proposes a wider time interval of 12–24 months postsurgery (2009, reconfirmed 2019) [11]. Since the publication of these guidelines, several studies have evaluated pregnancy course and neonatal outcomes in women who conceived at different time intervals after surgery, but results are often limited by small sample sizes. Furthermore, only a few studies evaluated the impact of gestational weight gain [9, 12, 13]. Therefore, the aim of this retrospective multicenter cohort study was to evaluate pregnancy and neonatal outcomes by surgery-to-conception interval and by adherence to the recommendations for gestational weight gain of the NAM.

## Materials and Methods

### Study Design

Data were extracted from medical records of female patients who previously underwent bariatric surgery and sought obstetric care at three large teaching hospitals in the Netherlands: Rijnstate hospital (Arnhem), Gelderse Vallei hospital (Ede), and Medical Centre Leeuwarden (Leeuwarden). Ethical approval for this study was obtained from all local institutional ethics committees.

All surgeries were performed between 2005 and 2018 and included Roux-en-Y gastric bypass (RYGB), one anastomosis gastric bypass (OAGB), and sleeve gastrectomy (SG). All deliveries occurred between October 2007 and August 2019. Exclusion criteria were spontaneous abortions, elective termination of pregnancy, multiple births, pre-existing diabetes mellitus, and insufficient data about pregnancy course.

### Outcomes

All pregnancies were categorized based on (1) surgery-to-conception interval and (2) adherence to the NAM recommendations for gestational weight gain [7].

Time from surgery to conception was defined as the period in months between the date of surgery and the date of conception. The conception date was estimated as the “first day of last menstrual period + 2 weeks” or as “due date – 40 + 2 weeks” when the first day of the last menstrual period was unknown. Based on the surgery-to-conception time interval, patients were categorized into three groups: the early group (≤ 12 months), the middle group (12–24 months), and the late group (> 24 months). Gestational weight gain was calculated as the difference between late pregnancy weight and prepregnancy weight in kilograms. Prepregnancy weight was reported as weight at the first antenatal visit or self-reported weight before pregnancy. Late pregnancy weight was extracted from medical records 4 weeks before delivery, at the earliest. Subsequently, weight gain was classified as inadequate, adequate, or excessive according to the NAM recommendations (Table 1) [7].

**Table 1:** National Academy of Medicine Weight Gain Recommendations for pregnancy [7]

Prepregnancy BMI	Total weight gain, kg
Underweight (< 18.5 kg/m^2^)	12.5–18.0
Normal weight (18.5–24.9 kg/m^2^)	11.5–16.0
Overweight (25.0–29.9 kg/m^2^)	7.0–11.5
Obese (≥ 30.0 kg/m^2^)	5.0–9.0

*BMI* body mass index

Next to surgery-to-conception interval and gestational weight gain, primary outcome variables were gestational age at delivery, preterm birth, birth weight, and weight-for-age percentile. Preterm birth was defined as < 37 weeks of gestation and very preterm birth as < 32 weeks of gestation according to the World Health Organization classification. Weight-for-age percentiles were calculated using the Dutch Perined birth weight charts, stratified for sex and gestational age at delivery in days [14]. Subsequently, LGA neonates (> 90th percentile) and SGA neonates (< 10th percentile) were identified.

Secondary outcome variables were Apgar score below 7 at 5 min, hospitalization of the neonate after birth, congenital defects, and perinatal death. Cases of perinatal death were excluded for analyses of neonatal outcomes.

Additionally, pregnancy-related complications were examined including gestational diabetes mellitus (GDM; new-onset diabetes diagnosed by glucose monitoring), pregnancy-induced hypertension (new-onset hypertension, above 140/90 mmHg at two occasions), preeclampsia (hypertension and proteinuria), and postpartum hemorrhage (postpartum bleeding of ≥ 1000 ml).

### Statistical Analysis

Differences in prepregnancy characteristics according to surgery-to-conception interval and gestational weight gain were analyzed by using one-way ANOVA for continuous data and chi-square tests for discrete data. Pregnancy and neonatal outcomes were analyzed by using multiple linear and logistic regression models while adjusting for maternal age, gravidity, parity, smoking status, prepregnancy BMI, and type of surgical procedure. The early group and the adequate weight gain group were used as reference groups.

These analyses were performed on individual pregnancies, which made it possible for a woman to contribute more than one pregnancy. Therefore, a sensitivity analysis was performed by the generalized estimating equation method with the mother’s identification number as a cluster and assuming an exchangeable correlation structure to adjust for the possible dependence between pregnancies from the same mother. In another sensitivity analysis, inclusion was restricted to the first pregnancy per woman (exclusion of 33 pregnancies). All statistical analyses were performed using SPSS Statistics version 25.0 for Windows (Armonk, NY: IBM Corp. 2017), and *P* < 0.05 was considered statistically significant. *P* values of planned pairwise comparisons with the reference groups were corrected by using the Bonferroni method.

## Results

### Demographic Characteristics

A total of 196 singleton pregnancies of 163 women who previously underwent bariatric surgery were included. The majority of the study population had a Caucasian ethnicity (87.8%). The most commonly performed bariatric procedure was RYGB (68.4%), followed by SG (23.5%) and OAGB (8.2%). The mean total body weight loss from surgery to conception was 30.9%, and about half of the women were still obese (BMI ≥ 30 kg/m^**2**^) at the time of conception. There were a few women with pre-existing hypertension (5.1%).

### Pregnancy and Neonatal Outcomes According to Surgery-to-Conception Interval

Table 2 shows prepregnancy characteristics and pregnancy and neonatal outcomes according to the surgery-to-conception interval. All groups were similar on prepregnancy characteristics, except for maternal age, prepregnancy BMI, and type of surgical procedure (*P* < 0.05 for all).

**Table 2:** Prepregnancy characteristics and pregnancy and neonatal outcomes according to time from surgery to conception

Outcomes	Early group (≤ 12 months, *n* = 46)		Middle group (12–24 months, *n* = 43)		Late group (> 24 months, *n* = 107)		*P* value	Pairwise comparisons
Prepregnancy characteristics								
Maternal age (years)	*28.9*	*± 5.0*	28.9	± 4.4	31.2	± 4.2	0.002	1 vs 3: *P* = 0.012
Type of surgical procedure							0.007	
RYGB	*26*	*(56.5)*	28	(65.1)	80	(74.8)		
OAGB	*5*	*(10.9)*	8	(18.6)	3	(2.8)		
SG	*15*	*(32.6)*	7	(16.3)	24	(22.4)		
Time from surgery to conception (months)	*7.6*	*± 3.5*	19.8	± 3.6	48.5	± 19.7	< 0.001	*P* < 0.001 for both
Prepregnancy BMI (kg/m^2^)	*32.8*	*± 7.6*	29.0	± 4.5	31.1	± 6.8	0.022	1 vs 2: *P* = 0.018
TBWL from surgery to conception (%)	*− 29.3*	*± 10.6*	− 34.2	± 9.0	− 30.2	± 11.5	*ns*	
Nulliparous	*22*	*(47.8)*	17	(39.5)	45	(42.1)	*ns*	
Smokers	*13*	*(28.3)*	7	(16.3)	23	(21.5)	*ns*	
Pre-existent hypertension	*4*	*(8.7)*	3	(7.0)	3	(2.8)	*ns*	
Pregnancy outcomes								
Gestational age (days)	*267.1*	*±19.9*	272.7	± 9.2	273.1	± 13.5	0.029	1 vs 3: *P* = 0.029
Preterm birth	*7*	*(15.2)*	2	(4.7)	9	(8.4)	*ns*	
Gestational weight gain (kg)^a^	*− 0.9*	*± 11.0*	+10.2	± 5.6	+10.0	± 6.4	<0.001	*P* < 0.001 for both
Adherence to the NAM recommendations^a^								
Inadequate	*30*	*(75.0)*	10	(24.4)	29	(32.6)	< 0.001	*P* < 0.001 for both
Adequate	*8*	*(20.0)*	17	(41.5)	25	(28.1)	*ns*	
Excessive	*2*	*(5.0)*	14	(34.1)	35	(39.3)	0.007	1 vs 2: *P* = 0.004 1 vs 3: *P* = 0.002
Cesarean section	*6*	*(13.0)*	4	(9.3)	25	(23.4)	*ns*	
GDM	*4*	*(8.7)*	3	(7.0)	9	(8.4)	*ns*	
Pregnancy-induced hypertension	*2*	*(4.3)*	3	(7.0)	6	(5.6)	*ns*	
Neonatal outcomes^b^								
Gender (male)	*23*	*(51.1)*	27	(64.3)	62	(58.5)	*ns*	
Birth weight (g)	*2979*	*± 470*	3161	± 481	3211	± 465	0.008	1 vs 2: *P* = 0.059 1 vs 3: *P* = 0.008
Weight-for-age percentile	*31.1*	*± 25.5*	35.5	± 27.0	37.7	± 28.9	*ns*	
LGA (> 90th percentile)	*1*	*(2.2)*	1	(2.4)	5	(4.7)	*ns*	
SGA (< 10th percentile)	*14*	*(31.1)*	11	(26.2)	20	(18.9)	*ns*	
Apgar score < 7 at 5 min	*2*	*(4.4)*	2	(4.9)	1	(0.9)	*ns*	
Admission at neonatology	*13*	*(28.9)*	7	(16.7)	22	(20.8)	*ns*	

Data are presented as mean ± SD or frequencies (percentages). Linear and logistic models are adjusted for maternal age, gravidity, parity, smoking status, prepregnancy BMI, and type of surgical procedure. *P* values of planned pairwise comparisons with the reference group (italicized) were corrected by using the Bonferroni method

*RYGB* Roux-en-Y gastric bypass; *OAGB* one anastomosis gastric bypass; *SG* sleeve gastrectomy; *BMI* body mass index; *TBWL* total body weight loss; *NAM* National Academy of Medicine; *GDM* gestational diabetes mellitus; *LGA* large-for-gestational-age; *SGA* small-for-gestational-age

^a^Missing: *n* = 26

^b^Cases of perinatal death were excluded for analyses of neonatal outcomes (*n* = 3)

Pregnancy occurred within 12 months after surgery in 23.5% (early group), within 12–24 months in 21.9% (middle group), and after 24 months postsurgery in 54.6% of the pregnancies (late group). The mean time from surgery to conception was 7.6 ± 3.5 months, 19.8 ± 3.6 months, and 48.5 ± 19.7 months, respectively.

The mean gestational age was significantly lower in the early group compared to the late group (267.1 ± 19.9 days vs 273.1 ± 13.5 days, *P* = 0.029). There was also a trend towards more preterm births in the early group compared to the middle and late group (15.2% vs 4.7%, and 8.4%, *P* = 0.093), but pairwise comparisons were not statistically significant. The mean gestational weight gain was significantly lower in the early group compared to the middle and the late group (− 0.9 ± 11.0 kg vs 10.2 ± 5.6 kg, and 10.0 ± 6.4 kg, *P* < 0.001 for both). Subsequently, women in the early group had a higher risk of inadequate gestational weight gain compared to women in the middle and late group (75.0% vs 24.4%, and 32.6%, *P* < 0.001), whereas the risk of excessive weight gain was lower (5.0% vs 34.1%, and 39.3%, *P* = 0.007). The mean neonatal birth weight was significantly lower in the early group in comparison to the late group (2979 ± 470 g vs 3211 ± 465 g, *P* = 0.008), but there was no significant difference in the risk of SGA neonates.

No other differences in pregnancy and neonatal outcomes were found. In both sensitivity analyses, the results for gestational age were borderline significant (*P* < 0.10; data not shown).

### Pregnancy and Neonatal Outcomes According to Gestational Weight Gain

Data on late pregnancy weight was available for 170 pregnancies. Table 3 shows prepregnancy characteristics and pregnancy and neonatal outcomes according to adherence to the NAM recommendations for gestational weight gain. All groups were similar on prepregnancy characteristics, except for prepregnancy BMI and type of surgical procedure (*P* < 0.05 for both).

**Table 3:** Prepregnancy characteristics and pregnancy and neonatal outcomes according to adherence to the NAM recommendations for gestational weight gain

Outcomes	Inadequate (*n* = 69)		Adequate (*n* = 50)		Excessive (*n* = 51)		*P* value	Pairwise comparisons
Prepregnancy characteristics								
Maternal age (years)	30.3	± 4.5	*29.3*	*± 4.9*	30.2	± 4.2	*ns*	
Type of surgical procedure							0.035	
RYGB	40	(58.0)	*39*	*(78.0)*	37	(72.5)		
OAGB	12	(17.4)	*2*	*(4.0)*	2	(3.9)		
SG	17	(24.6)	*9*	*(18.0)*	12	(23.5)		
Time from surgery to conception (months)	26.1	± 22.7	*30.9*	*± 20.3*	41.1	± 24.3	0.002	*ns*
Prepregnancy BMI (kg/m^2^)	32.7	± 8.7	*29.6*	*± 4.9*	29.9	± 4.3	0.020	2 vs 1: *P =* 0.040
TBWL from surgery to conception (%)	− 29.8	± 11.9	*− 33.1*	*± 9.9*	− 31.7	± 9.8	*ns*	
Nulliparous	36	(52.2)	*20*	*(40.0)*	22	(43.1)	*ns*	
Smokers	18	(26.1)	*8*	*(16.0)*	12	(23.5)	*ns*	
Pre-existent hypertension	7	(10.1)	*2*	*(4.0)*	1	(2.0)	*ns*	
Pregnancy outcomes								
Gestational age (days)	266.5	± 20.2	*273.8*	*± 8.4*	274.6	± 10.1	<0.001	2 vs 1: *P* = 0.002
Preterm birth	11	(15.9)	*3*	*(6.0)*	2	(3.9)	0.020	2 vs 1: *P* = 0.037
Cesarean section	13	(18.8)	*5*	*(10.0)*	12	(23.5)	*ns*	
GDM	10	(14.5)	*3*	*(6.0)*	1	(2.0)	*ns*	
Pregnancy-induced hypertension	6	(8.7)	*1*	*(2.0)*	3	(5.9)	*ns*	
Neonatal outcomes^a^								
Gender (male)	35	(52.2)	*31*	*(63.3)*	33	(64.7)	*ns*	
Birth weight (g)	3061	± 511	*3217*	*± 479*	3189	± 450	0.031	2 vs 1: *P* = 0.053
Weight-for-age percentile	37.1	± 29.4	*36.2*	*± 29.3*	35.5	± 25.7	*ns*	
LGA (> 90th percentile)	4	(6.0)	*2*	*(4.1)*	1	(2.0)	*ns*	
SGA (< 10th percentile)	17	(25.4)	*12*	*(24.5)*	10	(19.6)	*ns*	
Apgar score < 7 at 5 min	3	(4.5)	*1*	*(2.0)*	1	(2.0)	*ns*	
Admission at neonatology	16	(23.9)	*10*	*(20.4)*	11	(21.6)	*ns*	

Data are presented as mean ± SD or frequencies (percentages). Linear and logistic models are adjusted for maternal age, gravidity, parity, smoking status, prepregnancy BMI, and type of surgical procedure. *P* values of planned pairwise comparisons with the reference group (italicized) were corrected by using the Bonferroni method

*RYGB* Roux-en-Y gastric bypass; *OAGB* one anastomosis gastric bypass; *SG* sleeve gastrectomy; *BMI* body mass index; *TBWL* total body weight loss; *GDM* gestational diabetes mellitus; *LGA* large-for-gestational-age; *SGA* small-for-gestational-age

^a^Cases of perinatal death were excluded for analyses of neonatal outcomes (*n* = 3)

Gestational weight gain was adequate in only 29.4% of the pregnancies. It was inadequate in 40.6% and excessive in 30.0% of the pregnancies.

The mean gestational age at delivery was significantly lower in the inadequate weight gain group compared to the adequate weight gain group (266.5 ± 20.2 days vs 273.8 ± 8.4 days, *P* = 0.002). Additionally, there were more preterm births in the inadequate weight gain group (15.9% vs 6.0%, *P* = 0.037), among which all three very preterm births. The mean birth weight was also lower in the inadequate weight gain group (*P* = 0.031), but pairwise comparisons were not statistically significant, and there was no difference in the risk of SGA neonates.

When including only the first pregnancy after surgery, the results were similar compared to the primary analysis. In the other sensitivity analysis, the results for birth weight were not significant (data not shown).

### Pregnancy-Related Complications

The prevalence of pregnancy-related complications was low and not related to surgery-to-conception interval or gestational weight gain. GDM was most prevalent and occurred in 8.2% of the pregnancies (*n* = 16). In most cases, this could be treated by dietary management. Four women needed additional insulin therapy. Eleven women (5.6%) suffered from new-onset hypertension during pregnancy. None of them developed preeclampsia. Postpartum hemorrhage occurred in five cases (3.8%).

Congenital defects were observed in ten neonates (5.1%) and included congenital talipes equinovarus (clubfoot, *n* = 5), hypospadias (*n* = 2), anal atresia, syndactyly, and congenital hydrocephalus. There were three cases of perinatal death, one in each time group. During two of these pregnancies, gestational weight gain was inadequate. One neonate in the early and inadequate weight gain group was admitted to the neonatal intensive care unit because of a very preterm delivery (31 + 4 weeks).

Of the 150 pregnancies following a RYGB or OAGB, there were three cases of internal herniation. Two patients underwent successful laparoscopic closure of the internal herniation. The third patient, with a high suspicion of internal herniation at 27 weeks, experienced spontaneous resolution of symptoms and was therefore managed conservatively during pregnancy. Additionally, two women were admitted to the hospital because of gastrointestinal complaints and severe undernutrition and needed enteral nutrition.

## Discussion

Despite current recommendations, 23.5% of the women in this study cohort conceived within 12 months after bariatric surgery (early group). We found that gestational age at delivery, gestational weight gain, and neonatal birth weight were lower in this group than in the middle (12–24 months) and the late (> 24 months) group.

Overall, gestational weight gain was adequate in only 29.4% of the pregnancies. Inadequate weight gain during pregnancy was associated with lower gestational age at delivery and lower neonatal birth weight in comparison with adequate gestational weight gain. In addition, (very) preterm births were more frequently observed in this group.

Previous studies found no associations between the time from surgery to conception and adverse pregnancy or neonatal outcomes [4, 15–24]. In fact, most studies confirm that the risk of these outcomes is not increased during the first 12 months after bariatric surgery compared to later pregnancies [4, 16, 17, 19, 22, 25]. Nevertheless, we found that gestational age at delivery and neonatal birth weight were lower in pregnancies within 12 months postsurgery. Although the difference of ± 200 g in neonatal birth weight is probably not clinically relevant, the lower gestational age in the early group might be alarming as we also found a trend towards more preterm births in this group.

We also found that gestational weight gain was lower during the first 12 months after surgery. Weight gain during pregnancy may directly affect the immediate and future health of mother and child. Therefore, the NAM has published recommendations for adequate weight gain during pregnancy based on prepregnancy BMI [7]. In the present study, gestational weight gain was below the NAM recommendations in 75% of the women who conceived within 12 months and in 30% of the women who conceived after 12 months. Our results are in accordance with two other studies that also found that gestational weight gain was higher and more adequate when pregnancy occurred more than 12 months after surgery [19, 26].

Very few studies have addressed the risks of inadequate weight gain during pregnancy after bariatric surgery [9, 13]. In the current study, gestational weight gain was adequate in only 29.4% of all pregnancies. We found that inadequate gestational weight gain was associated with a lower gestational age at delivery. Moreover, we observed three times as many preterm births in this group, including all three very preterm births of < 32 weeks. In a large retrospective study including 337 pregnancies after RYGB, SG, and laparoscopic adjustable gastric banding, insufficient weight gain was a risk factor for preterm delivery when compared to excessive weight gain (adjusted OR, 6.40; 95% CI 2.41–17.0), but not when compared to adequate weight gain [13]. Furthermore, inadequate weight gain was associated with a lower birth weight in the present study. No differences were found for SGA or weight-for-age percentile, which is in line with findings from other studies [9, 27]. However, half of the women in the inadequate weight gain group even lost weight during pregnancy. In the systematic review and meta-analysis of Kapadia et al. [28], obese women with gestational weight loss had higher odds of SGA < 10th percentile (adjusted OR, 1.76; 95% CI 1.45–2.14) and SGA < 3rd percentile (adjusted OR, 1.62; 95% CI 1.19–2.20) compared to women with adequate weight gain.

We should encourage women who wish to conceive after bariatric surgery to avoid pregnancy until their weight has stabilized to minimize the risk of inadequate gestational weight gain. This is in line with the consensus recommendations of an international panel of experts [29]. Additionally, one should not underestimate the psychological impact of (gestational) weight gain in these women. In daily practice, we encounter many women who are afraid to gain weight during pregnancy. Healthcare professionals should be aware of the underlying factors and encourage these women to have adequate weight gain during pregnancy.

The prevalence of SGA (23%) was at least twice as high than what would be expected based on its definition (< 10th percentile) and higher than previously published data. The increased risk of SGA neonates is concerning since fetal growth restriction is associated with a higher risk of neonatal morbidity and mortality and the development of metabolic syndrome later in life [30, 31]. In order to break the vicious cycle of obesity and its health consequences, it is important that future research and clinical care focus on the prevention of SGA after bariatric surgery. On the other hand, we remarked a low prevalence of LGA as well as GDM and hypertensive disorders. Whereas obesity is a well-known risk factor for these outcomes, multiple studies found a decrease in LGA neonates, GDM, and pregnancy-related hypertensive disorders following bariatric surgery [2, 3].

This study is one of the largest series that evaluated pregnancy course and neonatal outcomes by surgery-to-conception interval and gestational weight gain. It should however be noted that the current sample size might have been too small for infrequent outcomes such as GDM and pregnancy-induced hypertension, increasing the risk of a type II statistical error. Another limitation is the retrospective nature of this study as the collected data depended entirely on the available data. Data on gestational weight gain were not always consistently registered throughout pregnancy, and patients’ prepregnancy weight may have been underestimated since they were predominantly self-reported. Moreover, data on nutritional deficiencies were limited and could not be included in the analyses. We therefore cannot exclude the possibility that these and additional unknown factors could have influenced the observed outcomes. Lastly, we combined data of pregnancies following different types of surgery as previous studies found no differences in pregnancy outcomes [32–34]. Despite including this factor into the statistical models, observed differences in the type of surgery could indicate an interrelationship between type of surgery, timing of pregnancy, and gestational weight gain. Larger, prospective studies are needed to confirm this trend.

## Conclusion

Our findings support the recommendation to postpone pregnancy for 12 months after bariatric surgery. During pregnancy, specific attention is needed on achieving adequate gestational weight gain. Future research should focus on the effect of inadequate gestational weight gain and maternal undernutrition on the duration of pregnancy and fetal growth, aiming to reduce the increased prevalence of SGA neonates after maternal bariatric surgery.
